# Potentiating Effects of MPL on DSPC Bearing Cationic Liposomes Promote Recombinant GP63 Vaccine Efficacy: High Immunogenicity and Protection

**DOI:** 10.1371/journal.pntd.0001429

**Published:** 2011-12-20

**Authors:** Saumyabrata Mazumder, Mithun Maji, Nahid Ali

**Affiliations:** Infectious Diseases and Immunology Division, Indian Institute of Chemical Biology, Jadavpur, Kolkata, India; René Rachou Research Center, Brazil

## Abstract

**Background:**

Vaccines that activate strong specific Th1-predominant immune responses are critically needed for many intracellular pathogens, including *Leishmania*. The requirement for sustained and efficient vaccination against leishmaniasis is to formulate the best combination of immunopotentiating adjuvant with the stable antigen (Ag) delivery system. The aim of the present study is to evaluate the effectiveness of an immunomodulator on liposomal Ag through subcutaneous (s.c.) route of immunization, and its usefulness during prime/boost against visceral leishmaniasis (VL) in BALB/c mice.

**Methodology/Principal Findings:**

Towards this goal, we formulated recombinant GP63 (rGP63)-based vaccines either with monophosphoryl lipid A-trehalose dicorynomycolate (MPL-TDM) or entrapped within cationic liposomes or both. Combinatorial administration of liposomes with MPL-TDM during prime confers activation of dendritic cells, and induces an early robust T cell response. To investigate whether the combined formulation is required for optimum immune response during boost as well, we chose to evaluate the vaccine efficacy in mice primed with combined adjuvant system followed by boosting with either rGP63 alone, in association with MPL-TDM, liposomes or both. We provide evidences that the presence of either liposomal rGP63 or combined formulations during boost is necessary for effective Th1 immune responses (IFN-γ, IL-12, NO) before challenge infection. However, boosting with MPL-TDM in conjugation with liposomal rGP63 resulted in a greater number of IFN-γ producing effector T cells, significantly higher levels of splenocyte proliferation, and Th1 responses compared to mice boosted with liposomal rGP63, after virulent *Leishmania donovani* (*L. donovani*) challenge. Moreover, combined formulations offered superior protection against intracellular amastigote replication in macrophages in vitro, and hepatic and splenic parasite load in vivo.

**Conclusion:**

Our results define the immunopotentiating effect of MPL-TDM on protein Ag encapsulated in a controlled release system against experimental VL.

## Author Summary

Visceral leishmaniasis (VL), a vector-transmitted disease caused by *Leishmania donovani*, is potentially fatal if left untreated. Vaccination against VL has received limited attention compared with cutaneous leishmaniasis, although the need for an effective vaccine is pressing for the control of the disease. Earlier, we observed protective efficacy using leishmanial antigen (Ag) in the presence of either cationic liposomes or monophosphoryl lipid A-trehalose dicorynomycolate (MPL-TDM) against experimental VL through the intraperitoneal (i.p.) route of administration in the mouse model. However, this route of immunization is not adequate for human use. For this work, we developed vaccine formulations combining cationic liposomes with MPL-TDM using recombinant GP63 (rGP63) as protein Ag through the clinically relevant subcutaneous (s.c.) route. Two s.c. injections with rGP63 in association with cationic liposomes and MPL-TDM showed enhanced immune responses that further resulted in high protective levels against VL in the mouse model. This validates the combined use of MPL-TDM as an immunopotentiator and liposomes as a suitable vaccine delivery system.

## Introduction

Leishmaniasis represents infections caused by obligate heterogenous kinetoplastid protozoan parasites, *Leishmania*, transmitted by the bite of infected female sand fly *Phlebotomus*. It afflicts hundreds of thousands of people in tropical and subtropical areas every year, is currently endemic in 88 countries, and poses a threat to 350 million people worldwide [1], [2]. The spectrum of leishmaniasis ranges from self-limiting cutaneous lesions to severe chronic mucocutaneous infections to fatal visceral disease. The latter is responsible for 59,000 death tolls every year, a parasitic disease statistics surpassed by malarial infections [3]. Conventional chemotherapies are often inadequate, toxic and expensive or are becoming less effective because of the emergence of resistance, a clear risk for human health. Extensive studies reveal that leishmaniasis is one of the parasitic diseases that can be controlled by vaccination [4]. Until recently, live virulent *L. major* (leishmanization) was the best vaccine against human cutaneous leishmaniasis (CL). However, this process was discontinued due to uncontrolled long-lasting skin lesions, the spread of HIV co-infection and the use of immunosuppressive drugs. Despite recent advances in immunology and determination of the factors that control the development of protective immune response in leishmaniasis, there are no available human vaccines against any form of leishmaniasis [5].

It is believed that persistence of a small number of parasites at the site of infection can trigger infection-induced immunity against CL [6]. This immunity requires the presence of leishmanial antigen (Ag) only rather than live replicating parasites [7]. An alternate way is to promote Ag exposure at the site of inoculation or to prevent Ag clearance using appropriate adjuvants like liposomes. Our group has recently demonstrated that stable cationic liposomes acted as a potent adjuvant to induce long-lasting protection against experimental visceral leishmaniasis (VL) [8], [9]. However, these results were obtained through intraperitoneal (i.p.) immunization and without the use of any immunomodulator. The major obstacle to the development of this vaccine for human use is the route of immunization. Since the route of vaccination influences the development of immune responses for protection, or failure of protection [10], the results obtained with i.p. immunization cannot be extrapolated to the clinically relevant subcutaneous (s.c.) route. Therefore to increase the prophylactic efficacy of liposomal protein vaccination through s.c. route against experimental VL, strategies are being attempted by choosing the best combination of adequate adjuvant with the vaccine delivery vehicle.

The traditional paradigm of s.c. immunization proposes involvement of skin derived dendritic cells (DCs), as biosensors, in Ag presentation that modulate the immune responses to the environmental stimuli. Despite the fact that delivery of liposomal Ag through s.c. route of immunization hindered the Ag uptake by draining lymph nodes (DLN) due to breakdown of liposomes in dermis [11], a cationic liposomal formulation with the synthetic mycobacterial immunomodulator (CAF01) exhibited substantial immune responses through activation of DCs against *Mycobacterium*, *Chlamydia*, and malaria [12]. Since the function of the liposomal formulation relied on the immunomodulator, successful liposomal vaccination would probably require an appropriate adjuvant along with the Ag delivery system. We chose cationic liposomal formulation with monophosphoryl lipid A (MPL) as the immunomodulator, a target for the intracellular Toll like receptor 4 (TLR4) with an extensive history of use in humans. MPL has been used as an adjuvant in several human clinical trials, including vaccines for tuberculosis, malaria, hepatitis B, and leishmaniasis [13]–[16].

However, the combinatorial use of immune potentiator with suitable delivery vehicle against VL has received minimum attention.

In the current study, we developed vaccination strategies using liposomal protein in association with MPL-TDM (monophosphoryl lipid A- trehalose dicorynomycolate) with proven vaccination efficacy through s.c. immunization in susceptible BALB/c mice against experimental VL. As the vaccine Ag we chose recombinant GP63 (rGP63) from *Leishmania donovani* whose native form has been shown to be highly protective against VL in BALB/c mice [8]. Here in this study, we analyzed the potentiating effects of distearoylphosphatidyl choline (DSPC)-bearing cationic liposomes in presence of MPL-TDM for the first time. To this end, we monitored the involvement of DCs in the antigen presentation for activation of effector T cells, leishmanicidal activity of macrophages and role of T cells in eliciting protective immunity. Additionally we examined the impact of MPL-TDM and liposomes on prime-boost.

## Materials and Methods

### Mice and parasites

Studies were performed with 4–6 weeks old female BALB/c mice reared in the animal care facility of the Indian Institute of Chemical Biology under pathogen free conditions. All animal studies were done according to the Committee for the Purpose of Control and Supervision on Experimental Animals (CPCSEA), Ministry of Environment and Forest, Govt. of India, and approved by the animal ethics committee (147/1999/CPSCEA) of Indian Institute of Chemical Biology. An Indian strain of *L. donovani* (MHOM/IN/83/AG83) was originally isolated from an Indian Kala-azar patient and maintained by serial passage in Syrian hamsters as described earlier [17]. The parasites were cultured as promastigotes at 22°C in Medium 199 (Sigma-aldrich, St. Louis, MO) supplemented with 2 mM glutamine, 25 mM HEPES, penicillin G sodium (100 U/ml), streptomycin sulphate (100 µg/ml) and 10% heat inactivated fetal bovine serum (FBS) (Gibco/BRL Life Technologies, Grand Island, USA). Parasites from stationary-phase culture were sub-cultured to maintain an average density of 2×10^6^ cells/ml.

### Generation of recombinant protein and entrapment in DSPC-bearing cationic liposomes

A plasmid containing full-length gp63 from *L. donovani* (pET16bLdgp63) was generated, expressed and purified as described previously [18].

Liposomal rGP63 was prepared by incorporation of rGP63 into the lipid bilayer of DSPC, cholesterol (Sigma-aldrich) and stearylamine (Fluka, Buchs, Switzerland) at a molar ratio of 7∶2∶2 and dissolved in chloroform followed by evaporating the organic solvents to form a thin film as described earlier [8]. Empty and Ag entrapped liposomes were prepared by dispersion of lipid film in either 1 ml of PBS alone or containing 250 µg/ml of Ag (rGP63). The mixture was then vortexed and sonicated in an ultrasonicator (Misonix, New York, USA) for 30 s, followed by incubation at 4°C for 2 h. The excess free rGP63 was removed by centrifugation at 100,000× g for 1 h at 4°C. The level of incorporation ranged between 65–70%.

### Immunization and challenge of infection

BALB/c mice were immunized s.c. two times at an interval of 2 weeks with 2.5 µg of rGP63 either free or entrapped within DSPC-bearing cationic liposomes, and or mixed with 25 µg of Sigma adjuvant system or MPL-TDM in a total volume of 100 µl. PBS, only liposomes, only MPL-TDM and liposomes mixed with MPL-TDM serve as controls. At 1 and 3 wks post-immunization, groups of mice were sacrificed for the analysis of immune response. In other experiments, mice were primed with liposomal rGP63 in association with MPL-TDM, followed by boosted with either rGP63 alone, or in association with MPL-TDM, liposomes or both. For experimental infections, mice were challenged 3 weeks post-immunization with 2.5×10^7^ freshly transformed stationary-phase promastigotes in 200 µl PBS injected via the tail vain as reported previously [17].

### Antigen presenting capacity of DC

Two days after immunization, DCs obtained from draining inguinal nodes were collected from the mice immunized with PBS, MPL-TDM plus liposome, and liposomal rGP63 plus MPL-TDM, and the Ag- presenting capacity was studied. In brief, lymph nodes (LN) were passed through 100 µm cell strainer and digested for 30 min at 37°C by 1 mg/ml of collagenase D (Roche Diagnostics, Mannheim, Germany). DCs were positively isolated by using magnetized anti CD11c^+^ magnetic-activated cell sorting columns (Miltenyi Biotec, Auburn, CA) according to manufacturers' protocol and the purity was simultaneously checked by flow cytometry. To detect the Ag presenting capability of DCs, CD4^+^ T cells were isolated from mice vaccinated with rGP63 through magnetized anti CD4^+^ antibody by positive selection. 2×10^5^ CD4^+^ T cells were co-cultured with 2×10^5^ CD11c^+^ and CD11c^−^ cells isolated from mice vaccinated with either liposomal rGP63 mixed with MPL-TDM, liposome plus MPL-TDM or PBS. Supernatants were collected 24 h later and assayed for IL-2 by sandwich ELISA using OptEIA kit (BD Pharmingen, San Diego, CA).

### Derivation and stimulation of bone-marrow derived dendritic cell (BMDC)

BMDCs were generated by culturing cells isolated aseptically from tibias and femurs of BALB/c mice in the presence of GM-CSF and IL-4 as reported previously [19]. Briefly, bone ends were cut and marrow flashed out with complete RPMI-1640, passed through a nylon mesh to remove small pieces of muscles and debri. Cells were pelleted by centrifugation at 390 g for 10 min and resuspended in complete RPMI medium containing rGM-CSF (40 ng/ml) and rIL-4 (30 ng/ml) supplemented with 10% FCS at a density of 1.5×10^6^/ml cells in 24 well plates. Two-thirds of the medium was replaced on day 4 of culture and maintained for additional 3 days inside 5% CO_2_ at 37°C. On day 8 of culture, most non-adherent and loosely adherent cells had acquired typical DC morphology and the phenotype was evaluated by flow-cytometry. On day 8, cells were collected, washed twice with ice-cold PBS, and adjusted to 1×10^6^ cells/ml in complete RPMI-1640 with 10% FCS and incubated in 24 well-plates with the adjuvants being tested. Supernatants were collected on days 1, 2, 3 and analyzed for IL-12 (p40) and Nitric oxide (NO).

### Measurement of delayed-type hypersensitivity (DTH) in mice

DTH was determined as an index of cell mediated immune response as described earlier [20]. The response was evaluated by measuring the difference of the footpad swelling at 24 h following intradermal immunization of the test footpad with 25 µl of rGP63 (200 µg/ml) from that of control (PBS-injected) footpad with a constant pressure calliper.

### Measurement of rGP63-specific antibody response

The levels of Ag-specific serum IgG and its different isotypes IgG1 and IgG2a was determined in serum samples from experimental mice before and after infection by ELISA. In brief, 96-well microtiter plates (Maxisorp, Nunc) were coated with rGP63 (5 µg/ml) diluted in 0.02 M phosphate buffer (pH 7.5) overnight at 4°C. The plates were blocked with 1% BSA in PBS at room temperature for 3 h to prevent non-specific binding. After washing with PBS containing 0.05% Tween-20 (Sigma), the plates were incubated with 1∶1000 dilutions of mice sera at 4°C. The next day, the plates were incubated for 3 h at room temperature with horseradish peroxidase-conjugated goat anti-mouse IgG1 or IgG2a diluted 1∶500 in blocking buffer. Substrate solution (0.8 mg/ml *o*-phenylene diamine dihydrochloride 0.05 M phosphate-citrate buffer pH 5.0 containing 0.04% H_2_O_2_) (100 µl) was added for 30 min. The absorbance was determined using an enzyme-linked immunosorbent assay (ELISA) plate reader (Thermo, Waltham, MA) at 450 nm.

### T cell proliferation assay and Cytokine ELISA

The spleen cells were aseptically removed from the immunized and infected BALB/c mice and single cell suspensions were prepared in RPMI 1640 supplemented with 10 mM NaHCO_3_, 10 mM HEPES, 100 U/ml penicillin, 100 µg/ml streptomycin sulphate, 50 µM 2-ME and 10% heat inactivated fetal bovine serum. Erythrocytes were removed by lysis with 0.14 M Tris buffered NH_4_Cl. The splenocytes were then washed twice, resuspended in the culture medium and viable mononuclear cell number was determined by Trypan blue exclusion [21]. Then the cells were cultured in a triplicate in a 96-well flat bottom plate at a density of 2×10^5^ cells/well in a final volume of 200 µl and stimulated with rGP63 (2.5 µg/ml). The supernatants collected were stored at −70°C for cytokine analysis. Measurement of IFN-γ, IL-2, IL-12(p40), IL-4, IL-10 levels was carried out as detailed in the instructions supplied with a cytokine ELISA kit (BD Biosciences).

### Measurement of nitric oxide (NO) production

The accumulation of nitrite in the culture medium was measured as described previously [21]. Briefly, 100 µl of splenocyte culture supernatants were mixed with an equal volume of Griess reagent (1% sulfanilamide and 0.1% *N*-1-naphthylethylene diamine hydrochloride in 50% H_3_PO_4_) and incubated at room temperature for 10 min. Absorbance was then measured at 540 nm.

### Evaluation of macrophage infection

At 3 weeks after the last booster immunization, macrophages collected from peritoneal exudates of vaccinated mice were allowed to adhere to cover slips in 0.5 ml RPMI 1640 containing 10% FCS at 37°C in 5% CO_2_. Nonadherent cells were removed by washing with warm PBS after 2 h. Macrophages were infected with promastigotes on glass cover slips (18 mm^2^; 10^6^ macrophages per cover slip) in 0.5 ml of RPMI/10% FCS at a ratio of approximately 10 parasites/macrophage for 4 h. The unphagocytosed parasites were removed by warm PBS washing, and the infected macrophages were further incubated in complete medium for 72 h at 37°C in 5% CO_2_. The cells were then fixed in methanol followed by staining with Giemsa for determination of intracellular parasite numbers. Prior to fixation, culture supernatants were removed at 72 h and frozen at −70°C for cytokine analysis.

### Evaluation of parasite burden in liver and spleen

Parasite load was evaluated by limiting dilution assay (LDA) with slight modifications [22]. Briefly, a weighted piece of liver and spleen were isolated from mice and homogenized in complete Schneider's Drosophila Medium (Invitrogen, Grand Island, USA) containing 10% heat inactivated FCS and diluted with a same medium to a final concentration of 1 mg/ml. Five-fold serial dilutions of homogenized tissues were cultured in a 96 well tissue culture plate (Nunc, Rosklide, Denmark) for one month at 22°C. The reciprocal of the highest dilution that was positive for parasite growth was considered to be the concentration of parasites per mg of tissue. The total organ burden was calculated using the weight of the respective organs.

### Flow cytometry

For intracellular analysis of IFN-γ produced by CD4^+^ and CD8^+^ T lymphocytes of vaccinated and infected mice, flow-cytometry (FACS Canto, BD Biosciences) using FACS Diva Software was carried out. All the antibodies were purchased from BD pharmingen. Single-cell splenocyte suspensions were stimulated overnight with 5 µg/ml rGP63 or left unstimulated. Brefeldin A (10 µg/ml) was added to the cultures 2 h before harvest. The cells were then washed in PBS containing 0.1% NaN_3_ and 1% FCS at 4°C and stained with PE-conjugated anti-CD3, PerCP Cy5.5 conjugated anti-CD4 and FITC-conjugated anti-CD8 mAb at 4°C for 30 min. The cells were then permeabilized with Cytofix/Cytoperm (BD Biosciences) solution for 20 min at 4°C, and then stained with APC conjugated anti-IFN-γ mAb. After incubation at 4°C for 30 min in the dark, cells were washed with wash buffer and re-suspended in staining buffer prior to analysis.

### Statistical analysis

One-way ANOVA statistical test was performed to assess the differences among various groups. Multiple comparisons Tukey-Kramer test was used to compare the means of different treatment groups using the GraphPad InStat software. In some experiments two-tailed Student's *t* test was performed. A value of *p*<0.05 was considered to be significant.

## Results

### Subcutaneous immunization with cationic liposomal rGP63 and MPL-TDM generated early immune responses

We have previously shown that the i.p. administration of native GP63 in association with DSPC bearing cationic liposomes was sufficient to confer durable immunity against BALB/c mice challenged with virulent *L. donovani* [8]. To design effective strategies that are crucial in s.c. immunization, we developed and optimized suitable vaccine preparations using recombinant form of *L. donovani* GP63 adjuvanted with either MPL-TDM or cationic DSPC liposomes or both.

**Figure 1:**
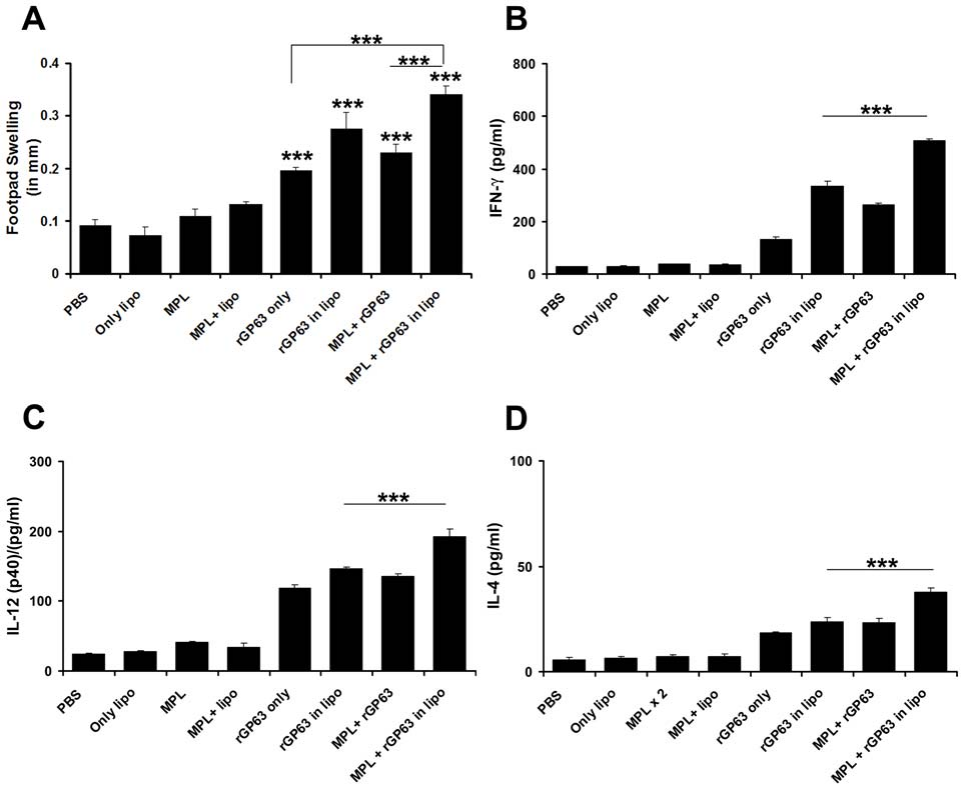
Induction of DTH and cytokine response in liposomal rGP63 plus MPL-TDM vaccinated mice. DTH and rGP63-specific cytokine analysis in mice vaccinated with either rGP63 alone, or in association with MPL-TDM, or DSPC-cationic liposomes, or both 3 weeks after final immunization. A. DTH response was determined as described in the Materials and Methods. Spleens were collected, splenocytes were restimulated in vitro for 72 h with 2.5 µg/ml of rGP63 and levels of (B) IFN-γ, (C) IL-12(p40), and (D) IL-4 were measured. All the results are mean ± S.E. of five individual mice per group, representative of two independent experiments with similar results. ***p<0.001 assessed by one-way ANOVA and Tukey's multiple comparison tests, compared to PBS, unless stated.

Mice were immunized with rGP63 alone or in association with either MPL-TDM, liposomes or both and the immune response was investigated by rGP63-specific immunity (Figure 1). Since DTH response is the measure of cell mediated immunity in vivo [20], we found mice receiving either rGP63 alone, in association with either MPL-TDM, liposomes or combined with both adjuvants showed significantly higher response compared to controls just 1 wk after final vaccination (data not shown) and maintained thereafter up to 3 weeks (Figure 1A). Mice vaccinated with liposomal rGP63 mixed with MPL-TDM, however, exhibited significantly higher level of DTH compared to mice receiving either rGP63 alone or mixed with MPL-TDM (p<0.001) (Figure 1A).

We next analyzed the cytokine responses in splenocytes isolated from vaccinated mice, following stimulation with Ag. The assays for IFN-γ, and IL-12 p40 production from immunized mice showed that vaccination with liposomal rGP63 mixed with MPL-TDM exhibited substantially higher immune response 1 week after final immunization (data not shown) and was further enhanced at 3 weeks post vaccination (Figures 1B and 1C). Moreover, mice vaccinated with liposomal rGP63 in association with MPL-TDM showed significantly higher IFN-γ and IL-12 p40 than the mice receiving rGP63 plus MPL-TDM or liposomal rGP63 (p<0.001). Interestingly, the IL-4 response was also clearly evident in the mice receiving rGP63 combined with two adjuvants and was significantly higher than the mice receiving rGP63 with MPL-TDM or liposomes (p<0.001) (Figure 1D).

In vitro analysis of mice sera obtained after the final immunization demonstrated that animals vaccinated with rGP63 adjuvanted with either MPL-TDM, or cationic liposomes or both generated enhanced levels of IgG2a (associated with Th1-biased response). Moreover, combining liposomal rGP63 with MPL-TDM resulted in a significantly high IgG2a compared to mice receiving MPL plus rGP63 or liposomal rGP63 (*p*<0.001) (Figure S1A). Although low level of serum IgG1 was observed in all vaccinated mice, maximum generation was observed in mice vaccinated with liposomal rGP63 alone, and combined with MPL-TDM (Figure S1B). These data suggest that s.c. immunization with rGP63 adjuvanted with combined adjuvants elicited significant cellular and humoral immunity at 3 weeks with early mixed Th1/Th2 immune response.

### Adjuvanting effects of DSPC-bearing cationic liposomes and MPL-TDM on BMDC activation

**Figure 2:**
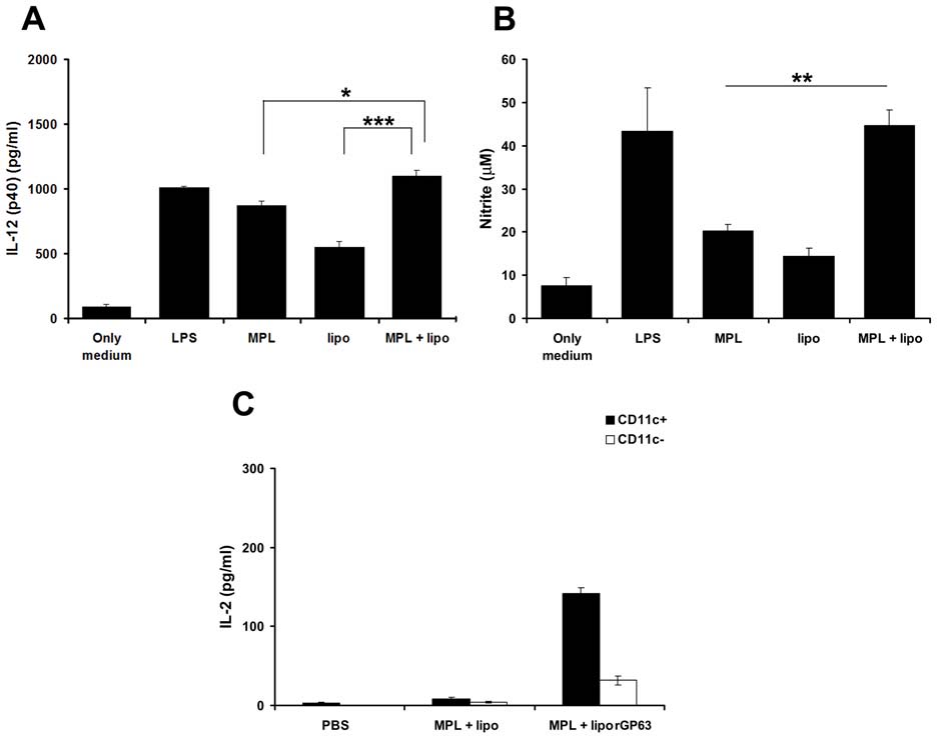
Activation of DCs in the generation of immune responses. A. Bone-marrow derived DCs were isolated and stimulated with LPS (1 µg/ml), liposomes (50 µM), MPL-TDM (100 ng/ml) and combination of liposomes (50 µM) and MPL-TDM (100 ng/ml) on activation of BMDCs. The release of IL-12 (p40) (A) and NO (B) were measured subsequently. The results are duplicate mean ± S.E, representative of three independent experiments with similar results. *p<0.05, **p<0.01, ***p<0.001 assessed by two-tail t test. C. Ag-presenting capacity of CD11c^+^ and CD11c^−^ cells isolated from mice immunized with PBS, MPL-TDM plus liposomes, and liposomal rGP63 plus MPL-TDM. CD11c^+^ and CD11c^−^ cells were isolated from LN as described in Materials and Methods and IL-2 content was assayed through ELISA.

It has been previously reported that TLR4 agonists like MPL can induce DCs to produce high levels of proinflammatory cytokines [23], [24], usually to a lower extent than its related compound LPS. To optimize formulations for prophylactic use, we investigated innate signals on DCs induced by MPL in presence or absence of cationic DSPC liposomes. Thus, we assessed the adjuvanticity of cationic DSPC liposomes, MPL-TDM and combined formulation (MPL-TDM plus liposomes) on DCs derived from the bone marrow of BALB/c mice. As shown in Figure 2A, culture with MPL-TDM, or cationic DSPC liposomes stimulated relatively high levels of IL-12 (p40). Interestingly, combining MPL-TDM with DSPC bearing cationic liposomes resulted in release of significantly higher levels (1100±43.3 pg/ml) of IL-12 (p40) compared to MPL-TDM (873.3±30.32 pg/ml) (p<0.05) or cationic DSPC liposomes (553.3±40.55 pg/ml) (p<0.001) and the response with the combinations was comparable to LPS (1013±7.26 pg/ml). Similarly, upon combined stimulation with MPL-TDM and cationic DSPC liposomes, the NO production from BMDCs was greatly increased (37.33±5.2 µM) and were significantly higher than MPL-TDM (25±2.8 µM) (p<0.01) and liposomes (22±2.64 µM) (p<0.01) (Figure 2B). These data indicated that combining MPL-TDM with cationic DSPC liposomes resulted in increased adjuvant activity on DCs in vitro. Taken together, these data illustrated the rational for using combined vaccine formulations to obtain the sustained immune responses in BALB/c mice.

### Ag presentation by lymph node dendritic cells (LNDCs)

DCs, the most potent APC of the immune system, play a crucial role in priming T cell immunity during *Leishmania* infection [25]. However, the activation of T cells by DCs depends on their state of processing, maturation and activation. Since the finding that s.c. immunization with cationic liposomal rGP63 in association with MPL-TDM demonstrated highest immune response after vaccination, we explored whether the early immune responses were due to activation of T-cells through draining node DCs. Naïve BALB/c mice were immunized s.c. with liposomal rGP63 mixed with MPL-TDM. Injections with PBS and liposomes mixed with MPL-TDM served as controls. We isolated both CD11c^+^ and CD11c^−^ cells from the LN draining the immunization site after 2d of immunization and co-cultured with CD4^+^ T cells isolated from mice immunized with liposomal rGP63 through i.p. route. Figure 2C illustrates that CD11c^+^ cells isolated from mice receiving liposomal rGP63 in combination with MPL-TDM, induced substantial amount of IL-2 from CD4^+^ T cells suggesting the ability of DCs to activate T cells. These data thus exemplified the role of DCs in the Ag presentation and concomitantly highlight the stimulatory effects of the vaccine comprising combined adjuvants on the immune system.

### Cellular and humoral responses prior *L. donovani* infection in mice primed with liposomal rGP63 and MPL-TDM, followed by boosting with either rGP63 alone, or in association with MPL-TDM, liposomes, or both

Based on the result obtained above, it appeared that above MPL-TDM in presence of liposomes contributed to the adjuvant effect of the liposomal rGP63 based protein vaccine. We further examined whether the combined adjuvant formulation was required for boosting to obtain maximal responses. To address this point, we developed several vaccine regimens in which priming was carried out using liposomal rGP63 in association with MPL-TDM, while boosting was either with rGP63 alone, in association with MPL-TDM, with DSPC liposomes, or both.

**Figure 3:**
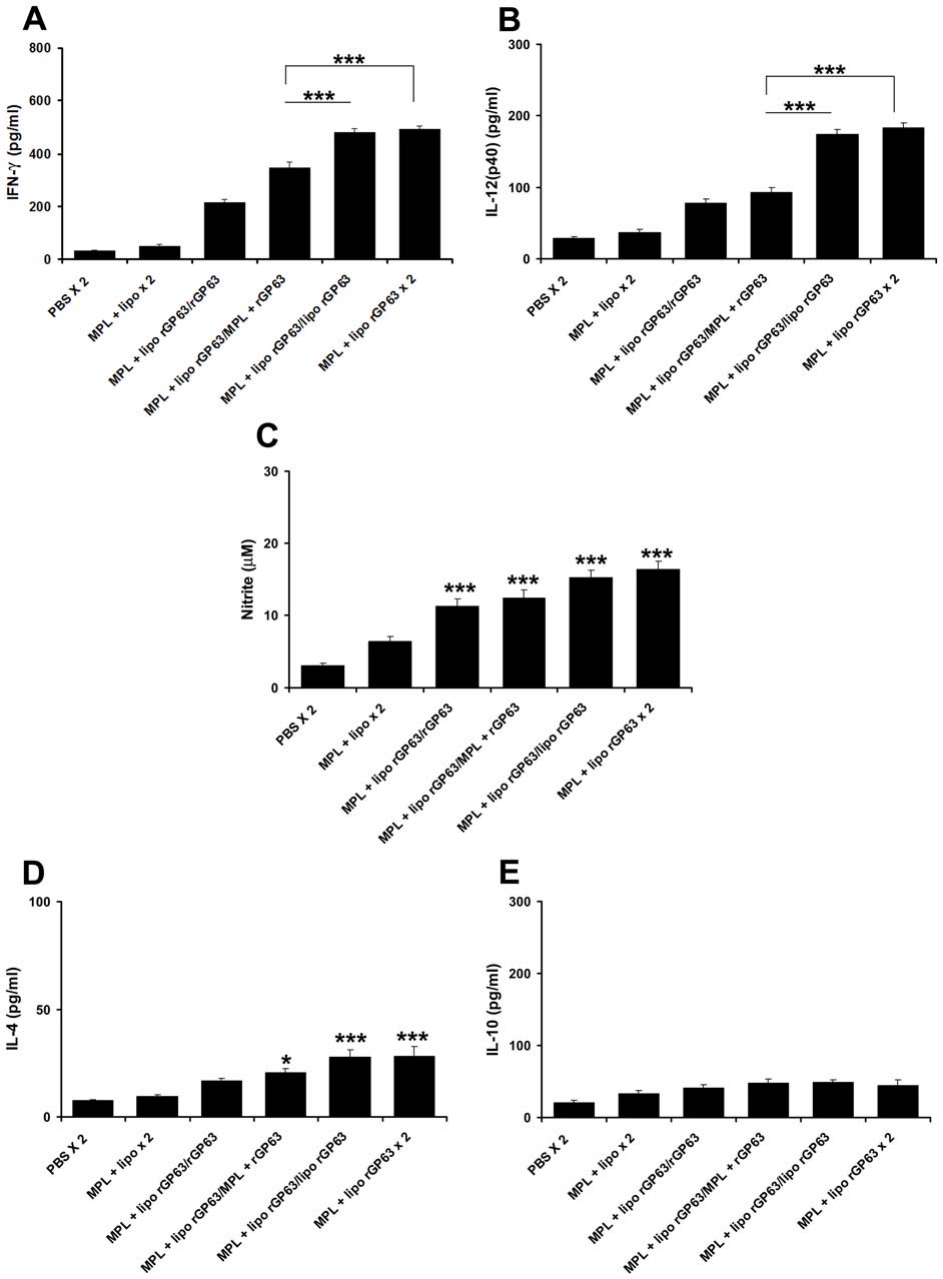
Cytokine and NO production in splenocytes from mice vaccinated with different prime/boost regimens. BALB/c mice primed with liposomal rGP63 plus MPL-TDM, followed by boosted with either rGP63 alone, in association with MPL-TDM, entrapped within liposomes, or both before *L. donovani* infection. rGP63-specific (A) IFN-γ, (B) IL-12 (p40), (C) NO, (D) IL-4, and (E) IL-10 were measured from cultured supernatants of splenocytes from vaccinated mice. The results are mean ± S.E. of five individual mice per group, representative of two independent experiments with similar results. *p<0.05, ***p<0.001 assessed by one-way ANOVA and Tukey's multiple comparison tests, compared to PBS, unless stated.

Boosting with rGP63 either with MPL-TDM, cationic liposomes or both showed comparable level of DTH, Ag-specific splenocyte proliferation, and IL-2 production (data not shown) 3 weeks after final vaccination. Significant enhancement of both the IgG2a and IgG1 isotypes with a dominance of IgG2a were observed in mice boosted with either rGP63 in association with MPL-TDM, or liposomes or both (Figure S2). However, mice boosted with liposomal rGP63 with (492.6±11.4 pg/ml IFN-γ, 183.2±6.8 pg/ml IL-12 p40) or without MPL-TDM (480.8±12.8 pg/ml IFN-γ, 174.2±6.46 pg/ml IL-12 p40) elicited comparable level of Th1 cytokine responses (IFN-γ, IL-12 p40) and the magnitude was significantly higher (p<0.001) than the group of mice boosted with rGP63 plus MPL-TDM (345±21 pg/ml IFN-γ, 93.2±6.1 pg/ml IL-12 p40) (Figures 3A and 3B) 3 weeks after final vaccination.

Furthermore, IL-4 response was significantly higher in mice boosted with liposomal rGP63 with or without MPL-TDM (Figure 3D) compared to PBS control (p<0.001). In addition, although highest level of NO was produced by splenocytes of mice boosted with liposomal rGP63 in association with MPL-TDM, the response was not significantly higher than mice boosted with liposomal rGP63 (Figure 3C).

**Figure 4:**
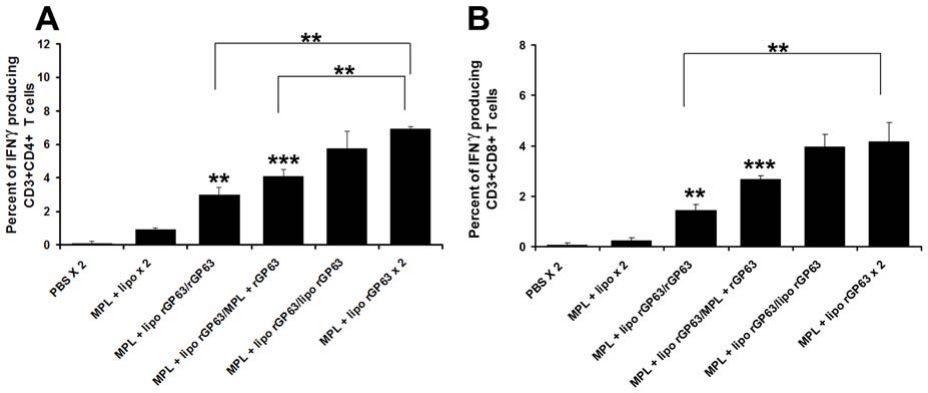
Flow cytometric analysis of rGP63-specific IFN-γ producing CD4^+^ and CD8^+^ T cells. BALB/c mice were primed with liposomal rGP63 plus MPL-TDM, followed by boosting with either rGP63 alone, in association with MPL-TDM, or liposomes, or both before challenge infection. Splenocytes were isolated from vaccinated mice and the expression of IFN-γ producing CD4^+^ (A) and CD8^+^ T (B) was studied in the presence of rGP63 (5 µg/ml). Data represent the mean of triplicate wells ± SE of three individual mice per group. **p<0.01, ***p<0.001 assessed by two-tail *t* test.

We next further compared and analyzed the IFN-γ producing CD4^+^ and CD8^+^ T cells in mice boosted with various vaccine potentials by intracellular cytokine staining through flow cytometry. Mice boosted with rGP63 alone showed significantly higher IFN-γ producing CD4^+^ (3.0±0.4%) as well as IFN-γ producing CD8^+^ T cells (1.4±0.2%) over PBS (p<0.01) immunized group (Figure 4). Moreover, mice boosted with rGP63 plus MPL-TDM showed significantly higher expression of CD4^+^ IFN-γ^+^ (4.1±0.3%; p<0.01) and CD8^+^ IFN-γ^+^ T cells (2.6±0.1%; p<0.01) compared to controls after 3 weeks post-vaccination. Interestingly, highest expression of IFN-γ producing CD4^+^ (6.9±0.1%) and CD8^+^ T cells (4.1±0.7%) was observed in mice boosted with MPL-TDM in combination with liposomal rGP63, which was comparable with mice boosted with liposomal rGP63 (CD4^+^ IFN-γ^+^ T cells 5.7±1%; CD8^+^ IFN-γ^+^ T cells 3.9±0.4%). Taken together, these results indicated that liposomal rGP63 either alone or in combination with MPL-TDM during boost seemed to modulate the immune system more efficiently than mice receiving rGP63 plus MPL-TDM during boost. Since boosting with liposomal Ag or liposomal Ag with MPL-TDM showed almost comparable post vaccination immune responses, the question we asked that whether MPL-TDM is required in boosting or not.

Because we observed that boosting with liposomal rGP63 either alone or in combination with MPL-TDM have accounted for comparable vaccine efficacy before *L. donovani* infection, we investigated whether this effect was also extended to experimental systems involving challenge infection with virulent *L. donovani*, an etiological agent of VL.

### Role of macrophages in antileishmanial activity

**Figure 5:**
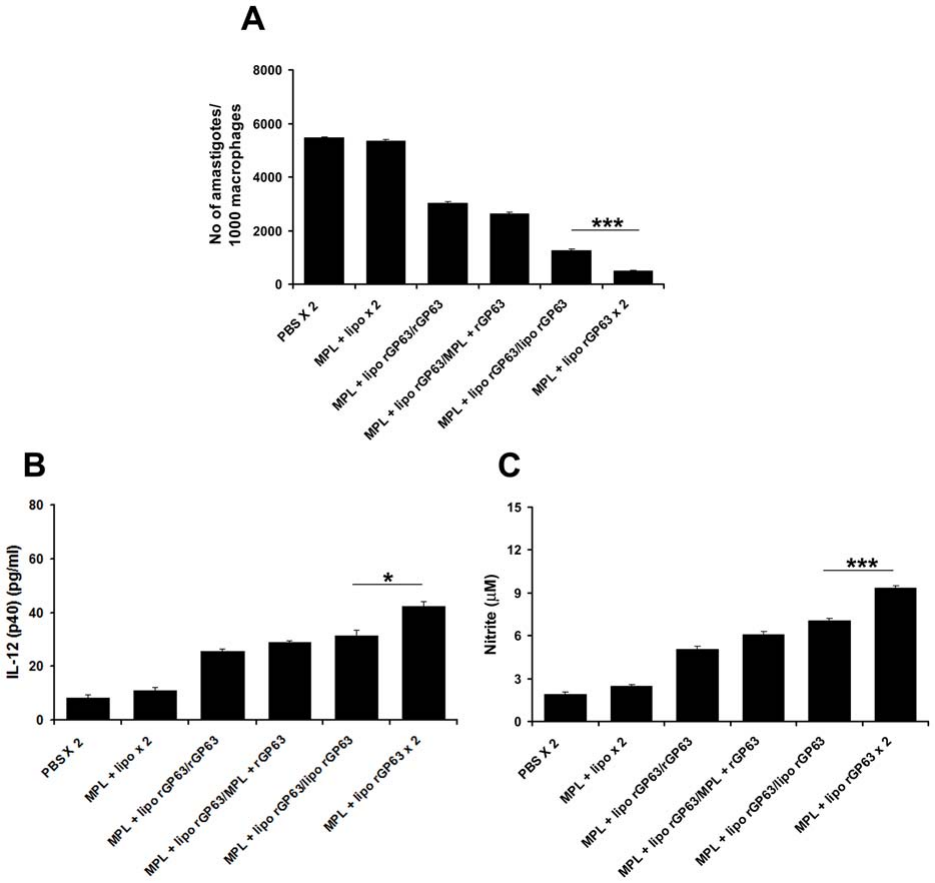
Determination of macrophage activation to suppress *L. donovani* infection. Peritoneal macrophages were isolated from different groups of vaccinated mice followed by in vitro infection with virulent *L. donovani*. The cells were stained with Giemsa for determination of intracellular parasite numbers (A). Culture supernatants were removed at 72 h and IL-12 (p40) (B), and the NO (C) production in the medium were estimated. Data represent mean ± SE of three individual mice per group. *p<0.05, ***p<0.001 assessed by Student's two-tail *t* test.

It is well established that activated macrophages from vaccinated mice inhibit *Leishmania* multiplication and arrest the parasite growth efficiently [26]. To assess the activation of antimicrobial responses elicited by macrophages to limit parasite multiplication, murine peritoneal macrophages were isolated from different prime-boost regimens and incubated with *L. donovani* promastigotes for 4 h. We observed that the numbers of infected macrophages from mice boosted with either rGP63 alone, entrapped within liposomes, in association with MPL-TDM or adjuvanted with both were significantly lower than the controls after 72 h infection (Figure 5A). Mean number of amastigotes per macrophage was significantly controlled in groups of mice boosted with liposomal rGP63 with MPL-TDM than mice receiving liposomal rGP63 alone. Thus, highest anti leishmanial activities were observed in mice receiving liposomal rGP63 and MPL-TDM during both prime and boost. Since stimulated macrophages produce IL-12 (p40) in response to intracellular pathogens, we investigated the production of this cytokine in supernatants of macrophages infected with parasite in vitro. Although detectable IL-12 (p40) was released from all the vaccinated mice, highest release of IL-12 was observed in mice receiving liposomal rGP63 in association with MPL-TDM (Figure 5B). The production of IL-12 (p40) in these macrophages correlated with the production of NO (Figure 5C). Collectively, these results indicated that boosting with liposomal rGP63 in presence of MPL-TDM showed highest action toward parasite elimination in *L. donovani* infected macrophages compared to mice boosted with liposomal rGP63 only.

### Evaluation of parasite burden

**Figure 6:**
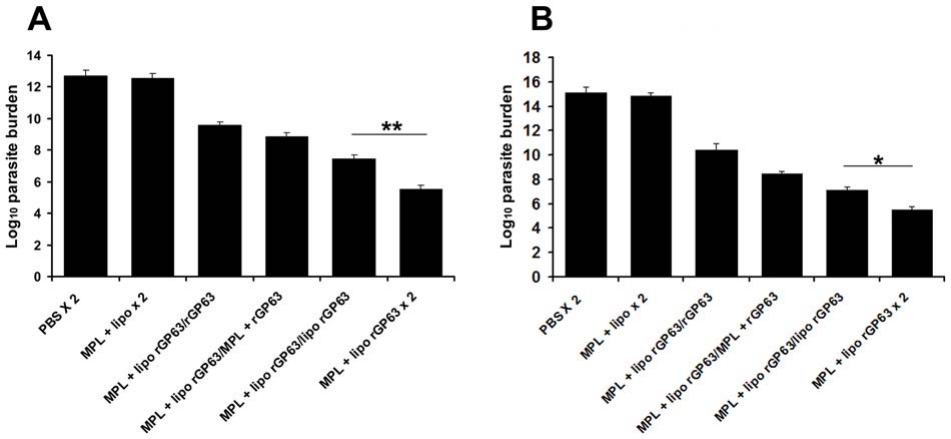
Evaluation of protection against *L. donovani* challenge in mice vaccinated with different vaccine regimens. Quantification of single viable cell was determined by limiting dilution assay performed 3 months after infection on the cells isolated from liver (A) and spleen (B). Results were expressed as log of total organ parasite burden. Data represent the mean ± S.E of five individual mice per group, representative of two independent experiments with similar results. *p<0.05, ** *p*<0.01 as assessed by one-way ANOVA and Tukey's multiple comparison test.

We next analyzed the hepatic and splenic parasite load in all the different vaccine regimens at 3 months post challenge measured by limiting dilution. The results showed that mice boosted with rGP63 adjuvanted with both cationic DSPC liposomes and MPL-TDM had ∼2 and ∼1.5-log-fold reduced parasite burden in liver and spleen respectively compared to mice boosted with liposomal rGP63 only (Figure 6). Furthermore, the data shown in Figure 6 indicated that both the formulations reduced parasite burden ∼5–7-log-fold in liver, and ∼7–9.5-log-fold in spleen, compared to unvaccinated mice. This extent of protection obtained so far has not been achieved in subunit protein based vaccination through s.c. route in susceptible BALB/c mice against VL.

### Assessing immune correlates of protective efficacy

To understand the mechanism underlying substantial and significant protection showed by the groups of mice receiving MPL-TDM in combination with liposomal rGP63 during prime-boost, we investigated the cellular and humoral responses after challenge infection with *L. donovani*, identifying cell types that can produce IFN-γ and cells responsible for sustained protection.

**Figure 7:**
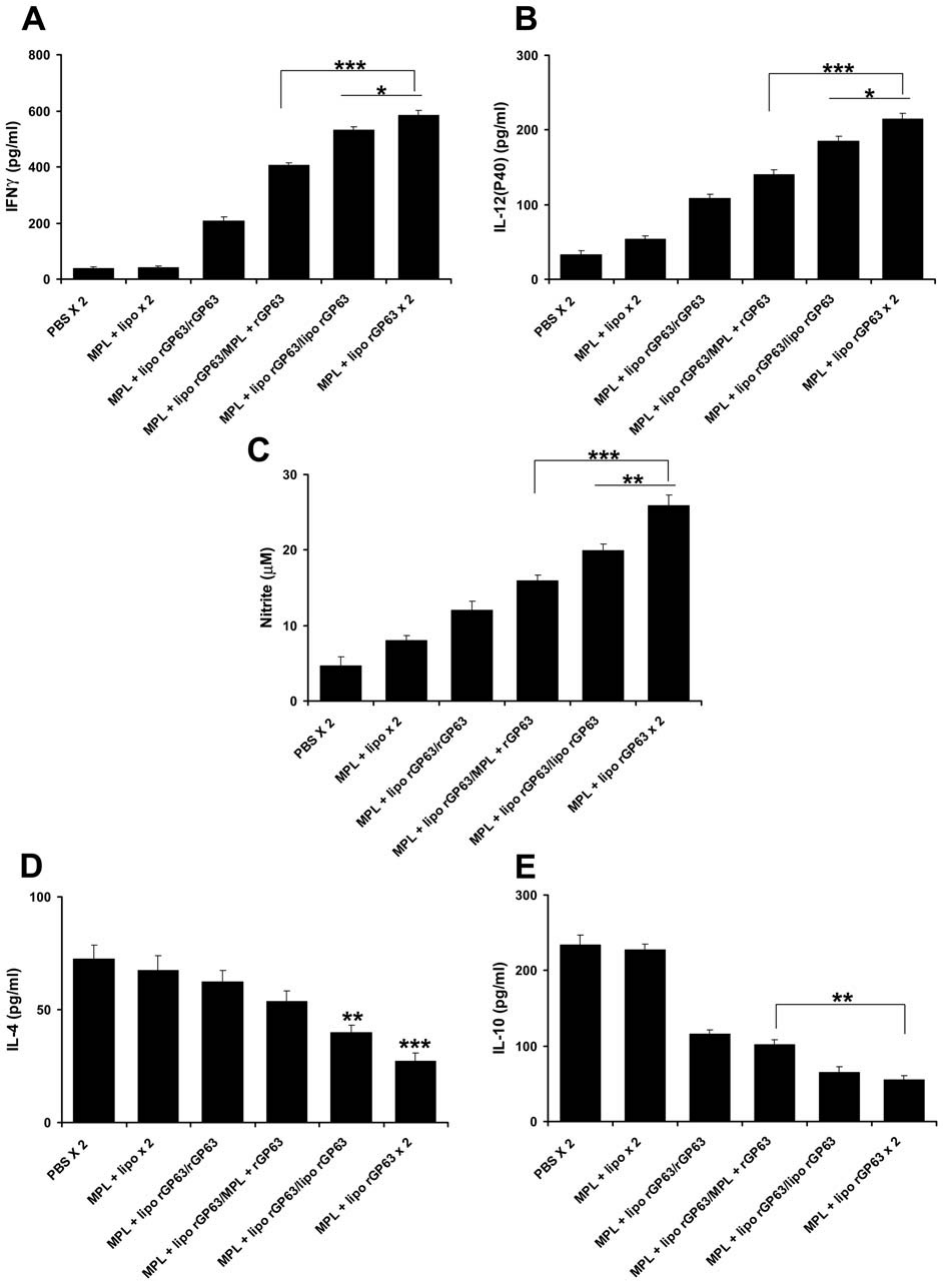
Cytokine and NO production in mice vaccinated with different prime/boost regimens 3 months post-infection with *L. donovani*. BALB/c mice were primed with liposomal rGP63 plus MPL-TDM, followed by boosted with either rGP63 alone, in association with MPL-TDM, entrapped within liposomes, or both after challenge with *L. donovani*. The cultured supernatants of splenocytes were collected, and assayed for (A) IFN-γ, (B) IL-12 (p40), (C) NO, (D) IL-4, and (E) IL-10. The results are shown as the mean absorbance values ± S.E of five individual animals per group, representative of two independent experiments with similar results. *p<0.05, **p<0.01, ***p<0.001 assessed by one-way ANOVA and Tukey's multiple comparison tests, compared to PBS, unless stated.

**Figure 8:**
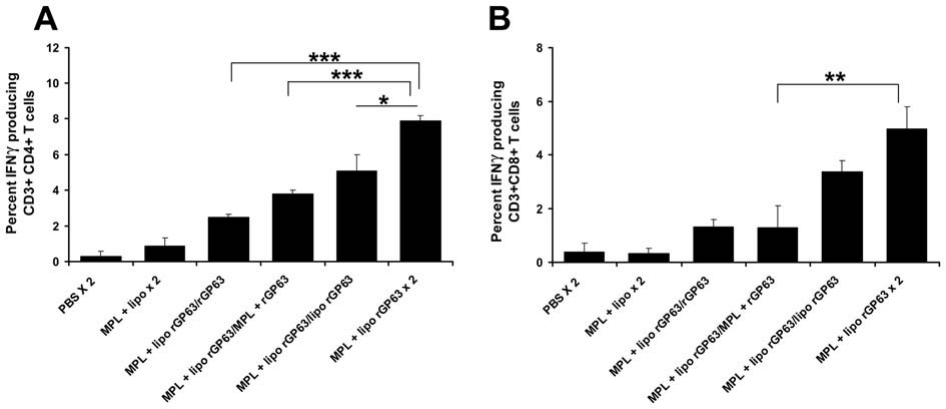
Flow cytometric analysis of rGP63-specific IFN-γ producing CD4^+^ CD8^+^ T cells after challenge infection. Mice were primed with liposomal rGP63 plus MPL-TDM, followed by boosting with either rGP63 alone, in association with MPL-TDM, entrapped within liposomes, or both after challenge with *L. donovani*. Splenocytes were isolated from vaccinated mice at 3 months after challenge infection and the expression of IFN-γ producing CD4^+^ (A) and CD8^+^ (B) T was studied in the presence of rGP63 (5 µg/ml). Data represent the mean of triplicate wells ± SE of three individual mice per group. *p<0.05, **p<0.01, ***p<0.001 assessed by Student's two-tail *t* test.

Since previous reports described that T cell proliferation is impaired during active VL [27], we explored Ag-induced T cell proliferation after infection (Figure S3). Although comparable levels of DTH and IgG isotype responses were observed in groups of mice boosted either with liposomal rGP63 alone or in association with MPL-TDM (data not shown), the proliferative responses were significantly higher in mice boosted with rGP63 and MPL-TDM compared to mice boosted with liposomal rGP63 (Figure S3). Strategies that favour Th1 response during infection have been shown to reduce *Leishmania* infection [28]. Moreover, induction of IFN-γ has been found to be involved in resistance to *Leishmania* infection in murine model [29], and is an essential upregulator of NO production by the macrophages. In contrast, IL-10 correlates with disease susceptibility [30]. Based on the important roles contributed to these cytokines, we investigated the levels of IFN-γ, IL-12 (p40), (Th1 cytokines) and IL-4, IL-10 (Th2 cytokines) after 3 months of infection. Our data suggested that boosting comprising combined adjuvants showed significantly higher IFN-γ responses (583.6±16.6 pg/ml) compared to boosting with liposomal rGP63 (531±11 pg/ml) alone after 3 months post infection (p<0.05) (Figure 7A). Since IL-12 is a potent inducer of Th1 cells and the control of *Leishmania* infection requires generation of strong Th1 response [31], we analyzed the IL-12 response in splenocytes of immunized mice after *L. donovani* infection. Figure 7B showed that mice boosted with liposomal rGP63 along with MPL-TDM exhibited significantly (p<0.05) highest IL-12p40 (214.6±7.7 pg/ml) compared to mice receiving liposomal rGP63 during boost (185.4±6.5 pg/ml). This response was also reflected in the generation of NO. There are significant differences in NO production between the groups of mice receiving either liposomal rGP63 alone or in combination with MPL-TDM at 3 months post-infection (Figure 7C). Moreover, combined adjuvant administration with rGP63 or mice boosted with liposomal delivery and Ag showed comparable levels of IL-4 production. These responses were significantly lower in comparison to unvaccinated mice (p<0.001) (Figure 7D). In contrast, the Th1 suppressive cytokine, IL-10, in the mice boosted with combined adjuvant formulations was significantly down-regulated than mice boosted with MPL-TDM plus rGP63 (p<0.01) (Figure 7E). Moreover, flow cytometric analysis showed 7.9±0.3% and 4.9±0.8% IFN-γ producing CD4^+^ and CD8^+^, respectively, in the *L. donovani* challenged liposomal rGP63 and MPL-TDM immunized mice during prime-boost, compared with 5±0.9% and 3.3±0.4% of IFN-γ producing CD4^+^ and CD8^+^, respectively, in the liposomal rGP63 boosted *L. donovani* challenged BALB/c mice (Figure 8). Therefore, mice boosted with liposomal rGP63 in association with MPL-TDM showed significantly higher IFN-γ producing CD4^+^ T cells compared to mice boosted with liposomal rGP63 (p<0.05) after 3 months *L. donovani* infection. Collectively, these results suggest that combined administration of cationic DSPC liposomes and MPL-TDM with rGP63 in a prime-boost regimen resulted in a significantly sustained Th1 biased immune response, which is extremely effective against *L. donovani* multiplication in susceptible BALB/c mice.

## Discussion

Despite the fact that the safety of protein-based subunit vaccination makes them highly attractive for human administration, a major limitation in the development of such vaccine remains the poor immunogenicity when used alone [32]. We believe that the development of protein-based vaccination could be greatly influenced by using suitable and effective adjuvant systems that de facto promote the slow release of Ag at the site of immunization with their ability to trigger the immune system. With the wide assortment of vaccine strategies that are immunologically effective, several studies have demonstrated that combining immunomodulators with controlled-release technologies led to potent and impressive immune responses [12], [33]–[36]. Toward this goal, our study shed new light on the approaches by using TLR4 agonist with liposomal delivery system, through s.c. route, in the susceptible BALB/c mouse model for translation of experimental results into humans. In the present study, we investigated the potentiating effects of MPL-TDM on cationic liposomal formulation against experimental VL.

In choosing effective immunomodulators, MPL is of interest based on its protective efficacy in experimental models of leishmaniasis [16], [37], [38], and safety and immunogenicity in humans [28]. MPL can activate the APCs through maturation of co-stimulatory molecules, and to secrete cytokines such as IL-6, IFN-γ, IL-12 which are crucial for activation and maturation of T and B cells [39]–[41]. Moreover, MPL-TDM was found to induce Th1 biased responses [42]–[44]. In our recent study, we demonstrated that vaccination with leishmanial Ag in combination with MPL-TDM elicited protective immune response against *L. donovani* challenge infection in BALB/c mice [45]. Therefore it is speculated that the effect of MPL-TDM might be enhanced in the presence of vaccine delivery system, such as liposomes, typically for the protein-based vaccination. Although immunogenicity of purified Ag is enhanced by cationic liposomal delivery [8], [9], [17], several studies showed that combination of a delivery vehicle and a Th1 inducing immunomodulator are prerequisite against *Leishmania* and tuberculosis [46]–[48]. Therefore, new generation vaccines are likely to be comprised of recombinant Ags used in conjunction with immunomodulators and delivery systems [49].

Developing effective vaccines against leishmaniasis is found to be difficult due to lack of appropriate vaccine adjuvants. Many attempts have been made to overcome this problem. Our earlier reports with leishmanial antigen (LAg) entrapped within DSPC liposomes showed protective efficacy against VL in BALB/c mice [21]. Moreover, cationic liposomes are commonly combined with immunomodulators to enhance the desired immune response towards Th1. Interestingly, soluble leishmanial antigen (SLA) in association with cationic liposomes and noncoding pDNA bearing immunostimulatory sequences elicited impressive protective responses against VL [36]. However, in all these studies, animals were immunized through i.p. route. In addition, recent studies have shown that s.c. vaccination with liposomal rGP63 partially protected susceptible BALB/c mice when challenged with *L. major* promastigotes [50]. Therefore, the immune response elicited by liposomal formulation could be modified by using MPL, a potent immunostimulator [51]. MPL signals via TLR4, which in turn activates the NF-κB and subsequent expression of pro-inflammatory cytokines. Since s.c. administration of MPL has been found to be successful against leishmaniasis [38], we used MPL as a potentiator of liposomal rGP63. Here, in this study, we evaluate the adjuvant role of MPL-TDM in liposomal rGP63 vaccine and compared the usefulness of these adjuvants in different prime boost regimens for the first time against VL.

Although strong adjuvanticity is prerequisite during priming, the impact of boosting is essential to study [52]. However, the use of heterologous prime-boost strategy in which immune response is primed with DNA followed by boosting with recombinant protein Ag has been successfully used in various diseases [53]. Since the prime-boost approach can improve the effectiveness of existing vaccines and quality of immune responses, several leishmanial Ags have been examined against experimental VL [54]. However, optimization of immune response of a vaccine can also be tested by the use of different adjuvants during prime/boost with same Ag. Interestingly, optimizing immune response by modulating vaccine components during boost is an essential approach to design effective vaccines [55]. In this study, we extended these findings by designing several vaccine formulations particularly during boost. Following priming with liposomal rGP63 in association with MPL-TDM, a strong immune response was observed. Although the adjuvant effect of MPL-TDM during boost was less than the cationic liposomes, the response was stimulated in combination with liposomal Ag delivery. While both the prime and boost influenced the immune response after immunization comparably, the ultimate difference was observed after challenge infection. Therefore, combination of delivery systems along with MPL is a novel approach for designing effective vaccines [51]. This adjuvant combination efficiently inhibited *L. donovani* multiplication in macrophages in vitro when administered during both prime and boost. Resistance to leishmaniasis is associated with a predominant IFN-γ and IL-12 production from the Ag-specific T cells [56], [57]. The results of the current study showed significantly higher levels of IFN-γ, and IL-12 (p40) in groups of mice receiving rGP63 in presence of liposomes and MPL-TDM during prime and boost than mice receiving liposomal rGP63 during boost, after *L. donovani* challenge infection. While MPL is reported to promote IFN- γ production by Ag-specific CD4^+^ T cells and skewing the immune response towards Th1 type [40], liposomal Ag showed protective response against leishmaniasis [21]. Because, NO is generated after macrophage activation by IFN-γ and plays an important role in controlling leishmaniasis [58], we measured the NO content in splenocytes of all vaccinated mice after *L. donovani* infection. Significantly higher NO was obtained in mice receiving combined adjuvant formulations than mice boosted with liposomal rGP63. To this end, the protective response showed that vaccination with liposomal rGP63 along with MPL-TDM showed almost 2-log-fold, and 7–10-log-fold reduction in parasite burden compared to mice boosted with liposomal rGP63 and unvaccinated mice, respectively. The ultimate impact of boosting was seen after challenge infection might be due to high Th1-biased response. Therefore, a clear requirement of all the vaccine components (rGP63, MPL-TDM, and liposomes) was evident for both priming and boosting. Until now, this extent of protection achieved using recombinant protein-based subunit vaccination formulated with MPL-TDM and a delivery system has not been achieved with any other subunit protein-based vaccination against experimental VL.

In conclusion, the advantage of simultaneous administration of liposomal Ag and TLR ligand through s.c. route is efficacious against experimental VL. This is the first report of using different prime/boost approach with TLR4 ligand along with liposomal delivery of subunit vaccination against experimental VL in susceptible BALB/c mice. This formulation resulted in activation of DCs, increased T cell response, higher IgG2a/IgG1 ratio resulting in superior protection against *L. donovani* infection.

## Supporting Information

Figure S1 **Liposomal rGP63 plus MPL-TDM induced antibody response.** rGP63-specific IgG isotype responses in mice immunized with either rGP63 alone, or in association with MPL-TDM, or DSPC-cationic liposomes, or both after immunization. 3 wks after last immunization, serum samples were collected and assayed for rGP63-specific (A) IgG2a and (B) IgG1 and (C) ratio of IgG2a/IgG1. The results are shown as the mean absorbance values ± S.E of five individual animals per group, representative of two independent experiments with similar results. **p<0.01, ***p<0.001 assessed by one-way ANOVA and Tukey's multiple comparison tests, compared to PBS, unless stated.
(TIF)

Figure S2 **Antibody response in different prime/boost regimens before challenge infection.** rGP63-specific IgG isotype responses in BALB/c mice primed with liposomal rGP63 plus MPL-TDM, followed by boosted with either rGP63 alone, in association with MPL-TDM, entrapped within liposomes, or both before challenge infection. 3 wks after last immunization, serum samples were collected and assayed for rGP63-specific (A) IgG2a and (B) IgG1 and (C) ratio of IgG2a/IgG1. The results are shown as the mean absorbance values ± S.E of five individual animals per group, representative of two independent experiments with similar results. *p<0.05, ***p<0.001 assessed by one-way ANOVA and Tukey's multiple comparison tests, compared to PBS, unless stated.
(TIF)

Figure S3 **rGP63-specific splenocyte proliferation after** ***L. donovani*** **infection.** Spleens were collected from vaccinated mice following 3 months post-infection, restimulated in vitro for 72 h with rGP63 (2.5 µg/ml). rGP63-specific splenocyte proliferation was determined by thymidine incorporation and expressed as counts per minute. The results are the mean values ± S.E of five individual animals per group, representative of two independent experiments with similar results. *p<0.05, ***p<0.001 assessed by one-way ANOVA and Tukey's multiple comparison tests.
(TIF)
