# Supplementary figures and images for: Potentiating Effects of MPL on DSPC Bearing Cationic Liposomes Promote Recombinant GP63 Vaccine Efficacy: High Immunogenicity and Protection
Source: PLoS Negl Trop Dis. 2011 Dec 20;5(12):e1429. doi: 10.1371/journal.pntd.0001429 (PMC3243702; doi:10.1371/journal.pntd.0001429)

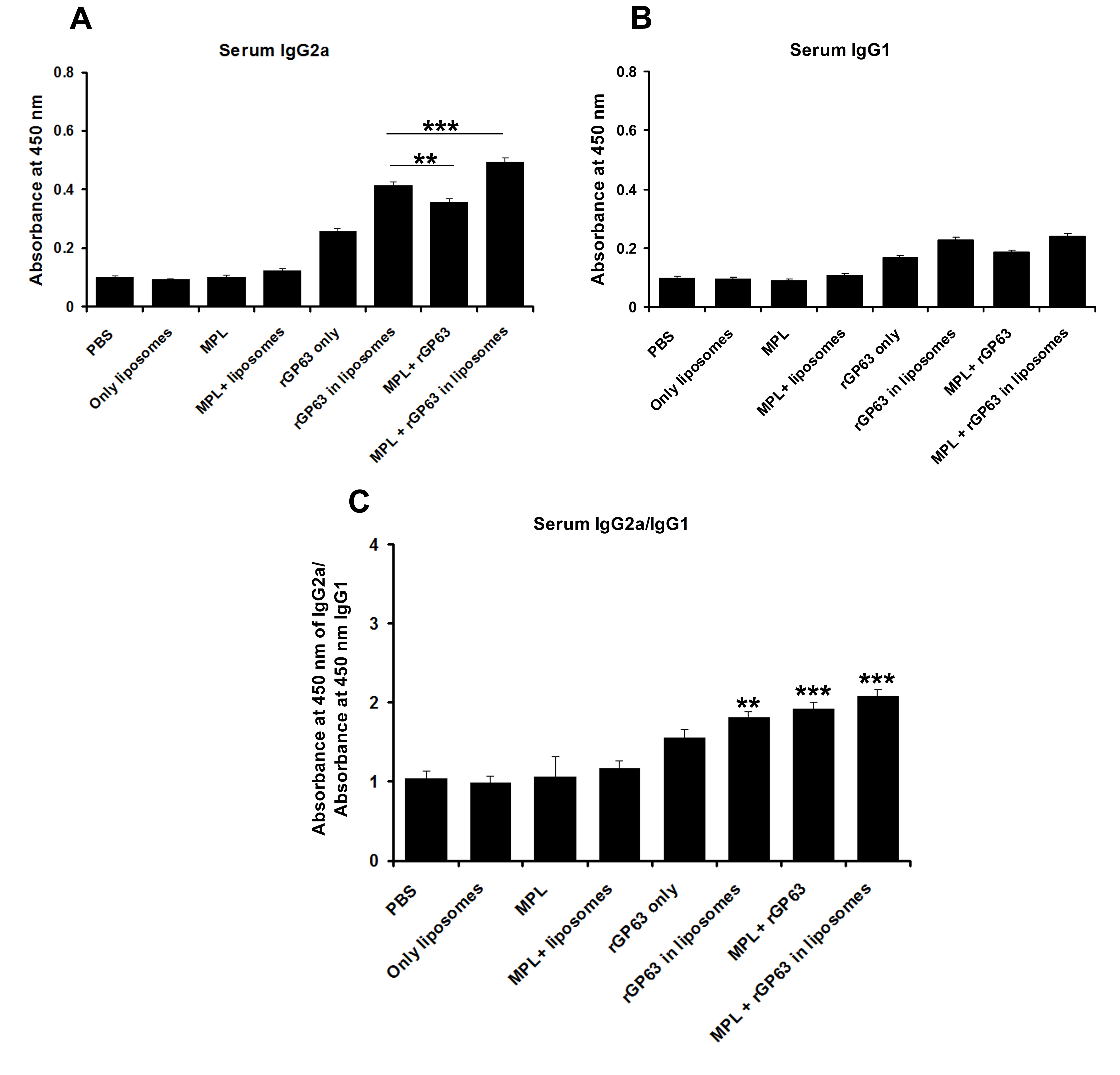

Supplement: Figure S1 — Liposomal rGP63 plus MPL-TDM induced antibody response. rGP63-specific IgG isotype responses in mice immunized with either rGP63 alone, or in association with MPL-TDM, or DSPC-cationic liposomes, or both after immunization. 3 wks after last immunization, serum samples were collected and assayed for rGP63-specific (A) IgG2a and (B) IgG1 and (C) ratio of IgG2a/IgG1. The results are shown as the mean absorbance values ± S.E of five individual animals per group, representative of two independent experiments with similar results. **p<0.01, ***p<0.001 assessed by one-way ANOVA and Tukey's multiple comparison tests, compared to PBS, unless stated. (TIF) [file pntd.0001429.s001.tif]

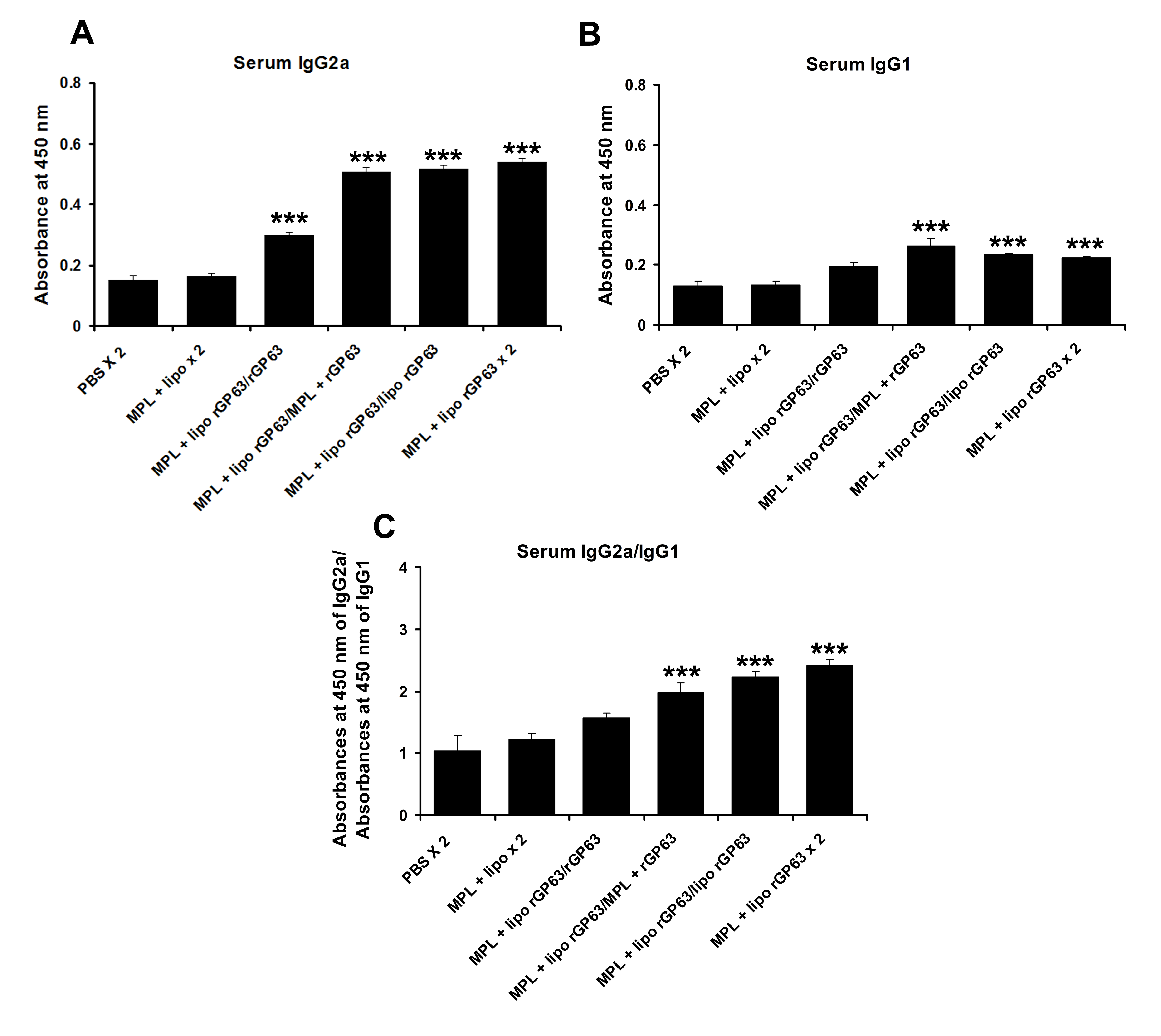

Supplement: Figure S2 — Antibody response in different prime/boost regimens before challenge infection. rGP63-specific IgG isotype responses in BALB/c mice primed with liposomal rGP63 plus MPL-TDM, followed by boosted with either rGP63 alone, in association with MPL-TDM, entrapped within liposomes, or both before challenge infection. 3 wks after last immunization, serum samples were collected and assayed for rGP63-specific (A) IgG2a and (B) IgG1 and (C) ratio of IgG2a/IgG1. The results are shown as the mean absorbance values ± S.E of five individual animals per group, representative of two independent experiments with similar results. *p<0.05, ***p<0.001 assessed by one-way ANOVA and Tukey's multiple comparison tests, compared to PBS, unless stated. (TIF) [file pntd.0001429.s002.tif]

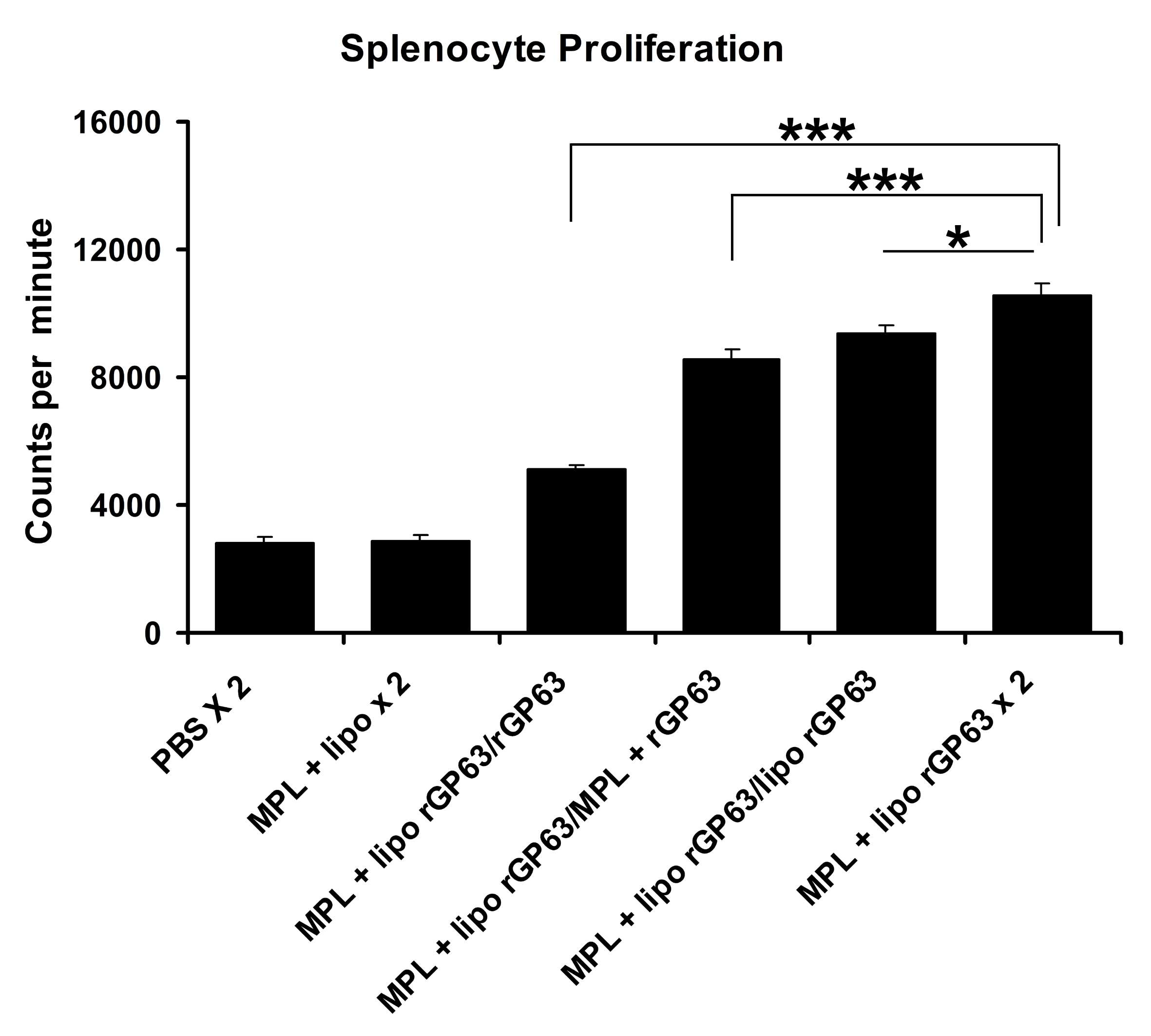

Supplement: Figure S3 — rGP63-specific splenocyte proliferation after L. donovani infection. Spleens were collected from vaccinated mice following 3 months post-infection, restimulated in vitro for 72 h with rGP63 (2.5 µg/ml). rGP63-specific splenocyte proliferation was determined by thymidine incorporation and expressed as counts per minute. The results are the mean values ± S.E of five individual animals per group, representative of two independent experiments with similar results. *p<0.05, ***p<0.001 assessed by one-way ANOVA and Tukey's multiple comparison tests. (TIF) [file pntd.0001429.s003.tif]
